# Physical Activity and Well-Being of High Ability Students and Community Samples During the COVID-19 Health Alert

**DOI:** 10.3389/fpsyg.2020.606167

**Published:** 2020-12-21

**Authors:** María de los Dolores Valadez, Elena Rodríguez-Naveiras, Doris Castellanos-Simons, Gabriela López-Aymes, Triana Aguirre, Juan Francisco Flores, África Borges

**Affiliations:** ^1^Institute of Psychology and Special Education, Department of Applied Psychology, University Center for Health Sciences, University of Guadalajara, Guadalajara, Mexico; ^2^Faculty of Social Sciences, Universidad Europea de Canarias, La Orotava, Spain; ^3^Transdisciplinary Research Center in Psychology, Autonomous University of the State of Morelos, Cuernavaca, Mexico; ^4^Department of Clinical Psychology, Psychobiology and Methodology, Faculty of Psychology and Speech Therapy, University of La Laguna, Santa Cruz de Tenerife, Spain

**Keywords:** psychological well-being, physical activity, Mixed Method Research, COVID-19 pandemic, high ability students

## Abstract

The health alert caused by the COVID-19 pandemic and the lockdown have caused significant changes in people’s lives. Therefore, it has been essential to study the quality of life, especially in vulnerable populations, including children and adolescents. In this work, the psychological well-being, distribution of tasks and routines, as well as the physical activity done by children and adolescents from two samples: community and high abilities, have been analyzed. The methodology used was Mixed Method Research, through a survey conducted online through social networks. The informants were the parents of the children and adolescents, 209 in the community sample and 116 in the high ability sample. The instrument used was a questionnaire implemented through Google Forms, with open and closed questions, including the Kidscreen-27 scale to measure well-being. The assessment of the adequacy of the physical activity levels was analyzed through ALCESTE. The results showed the absence of differences between students from community samples and those with high capacities in well-being and physical activity. Parents residing in Spain observed less play time in the high ability sample, and more time spent on homework, whether or not they have a diagnosis of high ability. It is concluded that these results question the misconceptions held about high ability students in terms of poorer personal adjustment and better interest in physical activities.

## Introduction

The health alert caused by the COVID-19 pandemic, which has forced people to remain in their homes, making it impossible to live normally, has had important effects at the psychological level, giving rise to emotions such as fear (Asmundson and Taylor, 2020), panic, anxiety or depression (Bao et al., 2020; Rajkumar, 2020). On the other hand, children seem to be more vulnerable when they experience traumatic events that disrupt their daily lives (Barlett et al., 2020), can react to the emotional states of adults (Centers for Disease Control and Prevention., 2020; Dalton et al., 2020). However, there are conflicting positions bout this issue: while Enmienda et al. (2020) consider that conditions of social isolation and confinement, together with mandatory online educational training may increase adverse feelings, Boonstra (2020) points out that anxiety is similar to that which may be exhibited by the general population.

A specific population to be analyzed in the health emergency caused by COVID-19 is high ability students, since following the theory that says they show high sensitivity, they may be more likely to present anxiety or depression (Karpinski et al., 2018; Amstrong et al., 2019). High capacities is not an easy construct to define, there is no definition shared by all experts (López Andrada et al., 2000; Colangelo and Davis, 2003; Pérez, 2006; Matthews and Yun Dai, 2014). Additionally, high capacity children and adolescents do not constitute an heterogenous group. Although the literature shows superior cognitive functioning than that observed in their less talented peers (Sastre-Riba, 2008; Sastre-Riba and Ortiz, 2018).

High intellectual capacity is the result of the expression of a high neurobiological potential of intellectual capacities, modulated by intra- and interpersonal variables along the evolutionary development (Olzewski-Kubilius et al., 2015). Intellectual capacity has been and still is one of the important variables in the definition of high capacity together with other variables (Fernández et al., 2017).

As far as the academic field is concerned, myths are especially related with the idea that high capacity means high performance. This idea is clearly mistaken, since it is a fact that the performance of high ability students is very often below what would be expected based on their abilities, giving rise to an important line of research under the label of underachievement (Gallagher, 1991; Hoover-Schultz, 2005; Dai et al., 2011; Steenbergen-Hu et al., 2020).

Stereotypes regarding lesser personal adjustment (Preckel et al., 2015; Baudson and Preckel, 2016; Matheis et al., 2017) have been shown to be false (Reis and Renzulli, 2004; Borges et al., 2011; Bacal, 2015; Valadez et al., 2015; Egeland, 2019; França-Freitas et al., 2019), or those relating to their satisfaction with life (Bergold et al., 2015).

Something similar occurs with psychological well-being, as studies show none or very small differences between gifted students and the control group (Zeidner and Shani-Zinovich, 2011; Olivier et al., 2016), or it depended on categories within the gifted group, where the creative gifted showed a lower degree of psychological well-being (Kroesbergen et al., 2016).

Household confinement has had other effects as well. Restricted mobility has reduced the possibility of exercise, which is a problem for the whole population, but it is particularly serious for children and adolescents, because of their need to move around (Hall et al., 2020; Hammami et al., 2020).

Physical activity is also important in the area of high ability, since one of the misconceptions that can be found is related to physical characteristics of high ability students, where it is common to consider them sickly, weak and not very likely to do physical activity and sports (Artiles and Jiménez, 2005; Guirado, 2015). The findings of a recent study on the difficulty of modifying beliefs showed that when the differences in the difficulty parameter are analyzed on a Likert-type scale using the item response theory in order to determine which statements are more easily modified, the idea that gifted pupils are weak and unhealthy seemed relatively easy to eliminate, but the myth that they are not very likely to be good sportsmen was among those that were more difficult to eliminate (Pérez et al., 2020).

However, the literature on physical activity among high ability students is quite limited, which makes it difficult to confirm or refute the idea that these students are not very interested in physical activity. There is relatively little research on how academically talented students occupy their time, as well as the place of sports and general physical activity in their free time. A study by Makel et al. (2015) comparing the distribution of time in and out of school for gifted students in the United States and India shows that time spent on extracurricular activities can vary depending on cultural and social variables, age, and family perceptions of the value of these activities [such as academics clubs, arts, service clubs, and sports activities (athletics)]. The authors cite previous research (Makel et al., 2011), which confirm the findings of Rinn and Wininger (2007) in which they found a high rate of participation of American adolescents in sports activities, which contradicts the idea that they spend little time on activities other than academics.

Some studies focus on pointing out the importance of sport and physical activity. Vidosavljević and Krulj-Drasković (2013) point out that it is a joint task between the family and the school to determine the ideal exercise to do. Lutostanski (2018) proposal is along with the same lines.

In terms of empirical studies, there is research that points to possible limitations of this student body. Thus, Lutostanski (2018) points out that, while most children with high ability can be good athletes, others can have asynchronous development, with physical activities not being easy for them, resulting in the idea that they may be clumsy or unprepared for tasks that require coordination.

In a similar line, Litster and Roberts (2011) found in their meta-analysis describing characteristics of gifted students that they scored better than their peers of lesser ability in perceived academic and behavioral competence and in overall self-concept, but their score was lower in perceived athletic competence and in appearance.

An important variable is whether or not they play sports, since gifted people who play sports have a better self-concept about their physical abilities than those who do not (Rinn and Wininger, 2007). The study by Hormazábal-Peralta et al. (2018) found that adolescents with high abilities reported high physical activity levels measured in hours per week. In contrast, females showed less weekly physical activity and a greater tendency to be obese.

The aim of this study is to compare the perceptions and opinions of parents or caregivers of a community sample of children and adolescents against another with high abilities on psychological well-being, physical activity and sedentary lifestyles developed during the health crisis produced by the COVID-19 pandemic. It is hypothesized, based on the studies reviewed, that high ability students will not present less psychological well-being, nor will they show lower activity patterns than the comparison group (community sample). Nor is it expected that there will be significant differences depending on the country of residence.

## Materials and Methods

### Methodology and Design

Mixed methodology was used (Mixed Methods Research, MMR; Johnson and Onwuegbuzie, 2004; Denscombe, 2008). The data was collected through a cross-sectional design (Smith et al., 2016), including survey methodology and qualitative methodology, through open questions that allow a qualitative analysis.

### Participants

A total of 325 participants responded to the survey. The average age was 41.54 (SD = 7.36), with a range of 23 to 65 years. More mothers (279) than fathers (44) participated and only two of the informants were guardians of the minors, specifically family members in charge of the child. Table 1 presents the data regarding the educational level of the parents and caregivers of both samples (high abilities and community). The procedure for selecting the sample was convenience, contacting the high ability population through specific high-skill partnerships. The consideration of high ability students was made by the affirmative response of the parents to the question of whether their child had been diagnosed as high ability. The number of families with children of high ability was 116, compared to 209 in the community sample (control group).

**TABLE 1:** Educational level of parents and caregivers in the two samples.

	**Level of studies**					**Total**
	**No studies**	**Secondary**	**Degree**	**Postgraduate**	**Ph.D.**	

	**Community sample**					
Mother	3	61	70	32	7	173
Father	0	4	21	5	4	34
Caregiver	0	0	1	1	0	2

**High ability sample**							
Mother	2	29	48	25	2	106
Father	0	0	7	1	2	10

Table 2 shows the age and sex of the children by type of sample. They have been divided into three groups: up to 7 years old, from 8 to 11 years old and from 12 years old onward. This division is made because the Psychological Wellbeing test is only applied to children of 8 years old or more, dividing the following group from 12 years old, following Piaget’s classification (Piaget, 1968) which indicates the appearance of formal operations from this age onward.

**TABLE 2:** Descriptive variables of age and sex in the two samples.

		**Age**			
**Sample**	**Sex**	**Up to 7 years old**	**8–11 years old**	**12 years old or more**	**Total**
Communitarian sample	Male	17	51	41	109
	Female	22	45	33	100
High ability sample	Male	5	16	13	34
	Female	3	57	22	82

The average age of the community sample is 9.64 (SD = 4.06) and the average age of the high capacity sample is 9.92 (SD = 3.27). There are no significant differences in age (Welch’ test = −0.692; freedom degree (f.d.) = 282.633; *p*-value (*p*) = 0.489).

The relationship between the sex of the child and belonging or not to the high ability sample is significant (Phi = 0.220; *p* < 0.001). However, it is common for the number of males in the high ability sample to be greater than females (Jiménez, 2010). In Spanish state data^1^, the statistics show that while the percentage of males in school is 51% (4241,424 of the total of 8217,662), the male student body diagnosed with high ability is 65% (23,092 of the total student body diagnosed, 35,494).

The participants resided in Spain (55.4%), Mexico (36.6%), Panama (4.3%), Colombia (2.5%), Chile (0.9%), and Argentina (0.3%). At the time of data collection (May 2020), all these countries had mobility restrictions outside the home due to the pandemic. This sample of different nationalities has been used to check the cross-validity of the psychological well-being and physical activity of gifted students compared to their peers, given that this reality is not limited to a single country. For this reason, an international Spanish-speaking sample has been used.

There was hardly any incidence of having suffered from COVID-19 in the family, since a large majority (318, which represents 97.2%) reported no cases. Four families reported having a confirmed patient in the family unit, while in another four cases there was a suspicion that a family member had the disease.

### Instruments

The data was collected with a questionnaire containing the following sections (see Table 3): (a) descriptive data of the participants; (b) characteristics of the family and their dwelling; (c) physical activity done by the minor and his/her assessment; (d) activity done during the day by the minor.

**TABLE 3:** Questions included in the used questionnaire.

**1. Descriptive issues of the sample**
Person completing the questionnaire
Participant’s age
Educational level of the participant Country of residence
Child’s age
Sex of child

**2. Family and housing issues**
Cases of coronavirus in the immediate family
Does your home have space to exercise?

**3. Kidscreen-27 scales**
General health
Health
Mood and feeling
The child’s family life and free time
Friends
School

**4. Physical activity**
Do you think that your child’s physical activity is sufficient or insufficient on a weekly basis?
How many hours a week does he/she run?
How many hours a week does he/she jump?
How many hours a week do you play ball?
How many hours a week does your child ride a bicycle?
How many hours a week do you do yoga?
How many hours a week do you do ballet?
How many hours a week do you do gymnastics?
How many hours a week do you do another sport or physical activity?

**5. Routine activities during the day**
Number of hours in a typical day that you are sitting Number of hours in a typical day that you are lying down (less time spent sleeping)
Number of hours in a typical day you are playing
Number of hours in a typical day you are watching TV
Number of hours spent on school activities

**6. Assessment of the physical activity carried out by the child**
Do you think your child gets enough exercise?

In addition, the Kidscreen-27 Parent Questionnaire (Ravens-Sieberer et al., 2005) was included in the same questionnaire to measure the psychological well-being of the children. It consists of 27 items, which are answered on a Likert-type scale of five alternatives (from nothing to a lot), structured into five scales: physical activity, mood, family life, friends and school. The reliability of the instrument is 0.82. The construct validity of the instrument is satisfactory; the calculation of the factor analysis explains 56% of the variance and for the reliability of each of the five dimensions.

### Procedure

The questionnaire was made in electronic format with the Google Forms program. It was sent by email and through social networks (WhatsApp, Facebook, Twitter) during the month of May 2020.

Regarding the ethical aspects, this research has been approved by the Commission of Ethics of Research and Animal Welfare of the University of La Laguna (CEIBA) with the Registration Number: CEIBA2020-0396. In the questionnaire the corresponding information for the participants was exposed, gathered in the Organic Law 3/2018, of December 5th, of Protection of Personal Data and guarantee of the digital rights (BOE n° 294 of December 6th), guaranteeing the anonymity and the confidentiality of the data. Finally, the use of the Kidscreen-27 Parent Questionnaire (Ravens-Sieberer et al., 2005), in its Spanish version, whose use was authorized by the authors of the Spanish version of the instrument.

### Data Analysis

The reliability of the Kidscreen Parent Questionnaire-27 test for the present sample was determined using Cronbach’s alpha. The identification of univariate outliers was made using the box plot diagram and the multivariate outliers calculating the Mahalanobis distance. To respond to the hypothesis of differences in well-being between the group of high communities and the community sample pupils, a MANOVA has been made, being the independent variables the type of sample (high capacities versus community sample) and the country of residence (Spain, Mexico and other American countries), the dependent ones the scales that compose the Kidscreen-27 Parent Questionnaire (Ravens-Sieberer et al., 2005. To confirm the equality of the covariance matrices, the Box test was used, and the Equality of Error Variances using the Levene test. The test used for the verification of multivariate normality was Shapiro–Wilks. The hypothesis of the existing differences in both samples in terms of physical activity and sedentary life were established by contrasting ANOVA, being the independent variables the type of sample and the country of residence, and the effect sizes by $\eta_{\text{p}}^{2}$. The testing of homoscedasticity was done with the Levene test. All analyses were performed with the SPPS program v.21. The chosen significance threshold is 0,05.

For the qualitative analysis, the phenomenological discourse analysis method was used, which identifies the meanings of language through lexical analysis using the ALCESTE (Lexical Analysis of Co-occurrences in Simple Text Statements) software (Reinert, 2001). This software uses statistical procedures to extract essential information from a text, in such a way that it will receive essential information from itself, quantifying its strongest lexical structures, grouping the co-occurrence. This co-occurrence is the association by proximity of various words (nouns, adjectives or verbs) using the Chi-square statistic, with the aim of differentiating the most significant lexical world. The unit of analysis is the Elementary Context Unit (ECU), which corresponds to the idea of a sentence or a set of between 8 and 20 words (De Alba, 2004). One of the advantages of this approach is that it avoids the subjectivity involved in the construction of categories by the researcher, since the computer program establishes the connections using statistical procedures (Bauer, 2003).

## Results

### Quantitative Analysis

#### Identification of any Univariate/Multivariate Outliers

To confirm the presence of univariate extreme cases, the box and whiskers diagram was used, without obtaining extreme cases. To contrast the existence of multivariate extreme cases, the Mahalanobis distance was calculated. A probability less than 0.001 was not obtained in any case.

#### Psychological Well-Being

The assumption of normal distribution was tested using the Shapiro–Wilks test. Although the distributions were not normal (Table 4), both ANOVA and MANOVA are robust to moderate violations of normality, provided that the violation is created by asymmetry and not by outliers (Tabachnick and Fidell, 2007). In the same Table 4 are presented the results of Levene’s test.

**TABLE 4:** Shapiro–Wilk normality test and Levene test.

**Scale**	**Shapiro–Wilk**	**d.f.**	***p***	**Levene**	**d.f.**	**d.f.**	***p***
Health	0.968	278	0.000	1.249	5	271	0.286
Mood	0.975	278	0.000	0.450	5	271	0.813
Family	0.974	278	0.000	1.330	5	271	0.252
Friends	0.962	278	0.000	1.162	5	271	0.328
School	0.960	278	0.000	0.690	5	271	0.631

In order to analyze if there were significant differences in the scales of psychological well-being in relation to the type of sample (having or not high ability) and to country of residence, a MANOVA was carried out. Since the Box test of equality of covariance matrices was significant (Box *M* = 100.402; *F*_60_, _2596_._440_ = 1.577; *p* = 0.003), the statistic used was Pillai’s trace. It did not yield significant results for any of the main effects (Sample: Pillai = 0.32; *F*_5_,_217_ = 1.785; *p* = 0.116; $\eta_{\text{p}}^{2}$ = 0.032. Country: Pillai = 0.61; *F*_10_,_536_ = 1.691; *p* = 0.800; $\eta_{\text{p}}^{2}$ = 0.031 or for the interaction (Pillai = 0.47; *F*_10_,_536_ = 1.691; *p* = 0.800; $\eta_{\text{p}}^{2}$ = 0.031).

#### Physical Activity

For the activity analyses, the responses given by parents of all ages were taken into account, i.e., also including those younger than 8 years old. To check if there were extreme cases in the variables related to the physical activity levels, the box and mustache diagram was made. Based on that, 48 participants were selected as extreme cases and removed, and the activities of yoga and ballet were eliminated, due to the low frequency observed.

Families were asked whether their children’s activity during confinement was sufficient or insufficient. No relationship was shown between type of sample and satisfaction or dissatisfaction with the activity performed (Phi = −0.068, *p* = 0.260). Response frequencies are shown in Table 5.

**TABLE 5:** Perception of the adequate physical activity developed by their children.

	**Physical activity**		
**Sample**	**Insufficient**	**Sufficient**	***N***
Community	112	64	176
High ability	71	30	101
*N*	183	94	277

As for the conditions of the dwelling, that is, whether they had an outside space for exercises, the results are presented in Table 6. The variables of sample type and outdoor space are not related (Phi = −0.111; *p* = 0.064).

**TABLE 6:** Outdoor space in the home for exercise.

	**Outdoor space**		
**Sample**	**No**	**Yes**	***N***
Community	35	141	176
High ability	30	71	101
N	65	212	277

After removing extreme cases from the sample, the normality of the distribution was verified with the statistic of Shapiro–Wilk, observing absence of normality (Table 7), although the violation of normality does not imply a problem of great relevance (Tabachnick and Fidell, 2007).

**TABLE 7:** Shapiro–Wilk normality test and Levene test.

**Physical activity**	**Shapiro–Wilk**	**d.f.**	***p***	**Levene**	**d.f.**	**d.f.**	***p***
Runs	0.758	277	0.000	1.331	5	271	0.251
Jumps	0.370	277	0.000	3.479	5	271	0.005
Plays ball	0.770	277	0.000	1.035	5	271	0.398
Rides his/her bike	0.690	277	0.000	2.191	5	271	0.056
He/she does gym	0.606	277	0.000	9.422	5	271	0.000
He/she does sport	0.674	277	0.000	2.337	5	271	0.042

In order to determine if parents perceive significant differences in their children’s levels of physical activity according to the sample (diagnosed or not with high capacities) and the country of residence, ANOVA was carried out for the different activities. Results are shown in Table 8.

**TABLE 8:** ANOVA of physical activity by sample and country of residence.

	**Sums of squares**	**d.f.**	**Mean square**	***F***	***p***	**$\eta_{\text{p}}^{2}$**
**Runs**						
Sample	6.826	1	6.826	1.135	0.288	0.004
Country	14.288	2	7.144	1.187	0.307	0.009
Interaction	3.869	2	1.934	0.322	0.725	0.002
Error	1630.522	271	6.017			

**Jumps**						
Sample	0.035	1	0.035	0.164	0.686	0.001
Country	0.559	2	0.280	1.316	0.270	0.010
Interaction	0.500	2	0.250	1.178	0.310	0.009
Error	57.539	271	0.212			

**Plays ball**						
Sample	0.818	1	0.818	0.161	0.688	0.001
Country	4.635	2	2.317	0.457	0.633	0.003
Interaction	0.050	2	0.025	0.005	0.995	0.000
Error	1373.063	271	5.067			

**Rides his/her bike**						
Sample	0.359	1	0.359	0.135	0.714	0.000
Country	12.735	2	6.367	2.386	0.094	0.017
Interaction	0.927	2	0.464	0.174	0.841	0.001
Error	723.321	271	2.669			

**He/she does gymnastic**						
Sample	0.616	1	0.616	0.239	0.626	0.001
Country	63.683	2	31.841	12.327	0.000	0.083
Interaction	1.053	2	0.526	0.204	0.816	0.002
Error	700.035	271	2.583			

**He/she does sport**						
Sample	7.016	1	7.016	1.512	0.220	0.006
Country	0.888	2	0.444	0.096	0.909	0.001
Interaction	4.245	2	2.122	0.457	0.633	0.003
Error	1257.730	271	4.641			

As it can be seen, there are only significant differences in gymnastic activity between countries. Since heteroscedasticity has been proven, the existence of significant differences was tested by the Brown-Forsythe test (B-F_2_,_69_._92_ = 13.227; *p* > 0.001). The average time dedicated to gymnastics in Spain was 1.34 (SD = 1.85), 0.32 (SD = 1.12) in Mexico and 0.50 (SD = 1.75) in the rest of the American countries. Tukey’s test showed significant differences between Spain and Mexico (difference of 1.02, *p* > 0.001).

Regarding what activities they do during the day, we also calculated the normality of the scores (Shapiro–Wilks test) and the homogeneity (Levene’s test), which are shown in Table 9.

**TABLE 9:** Shapiro–Wilk normality test and Levene test.

**Activity**	**Shapiro–Wilk**	**d.f.**	***p***	**Levene**	**d.f.**	**d.f.**	***p***
Sitting	0.829	276	0.000	3.325	5	271	0.006
Lying down	0.733	276	0.000	3.952	5	271	0.002
Playing	0.940	276	0.000	0.428	5	271	0.829
Watching TV	0.893	276	0.000	1.536	5	271	0.179
Doing homework	0.892	276	0.000	16.425	5	271	0.000

To check if parents observed differences in the daily activities done by their children according to the country of residence and the type of sample, ANOVA tests were carried out. Results are shown in Table 10.

**TABLE 10:** ANOVA of the differences in activities they do during the day.

	**Sums of squares**	**d.f.**	**Mean square**	***F***	***p***	**$\eta_{\text{p}}^{2}$**
**Sitting**						
Sample	2.279	1	2.279	0.225	0.636	0.001
Country	25.997	2	12.998	1.282	0.279	0.009
Interaction	2.078	2	1.039	0.102	0.903	0.001
Error	2747.232	271	10.137			

**Lying down**						
Sample	29.931	1	29.931	3.514	0.062	0.013
Country	36.315	2	18.158	2.132	0.121	0.015
Interaction	2.644	2	1.322	0.155	0.856	0.001
Error	2308.085	271	8.517			

**Playing**						
Sample	16.286	1	16.286	4.521	0.034	0.016
Country	27.860	2	13.930	3.867	0.022	0.028
Interaction	26.048	2	13.024	3.615	0.028	0.026
Error	972.664	270	3.602			

**Watching TV**						
Sample	5.155	1	5.155	2.589	0.109	0.009
Country	4.857	2	2.428	1.220	0.297	0.009
Interaction	4.879	2	2.439	1.225	0.295	0.009
Error	539.520	271	1.991			

**Doing homework**						
Sample	0.892	1	0.892	0.157	0.692	0.001
Country	233.301	2	116.651	20.504	0.000	0.132
Interaction	4.653	2	2.326	0.409	0.665	0.003
Error	1536.044	270	5.689			

**FIGURE 1:**
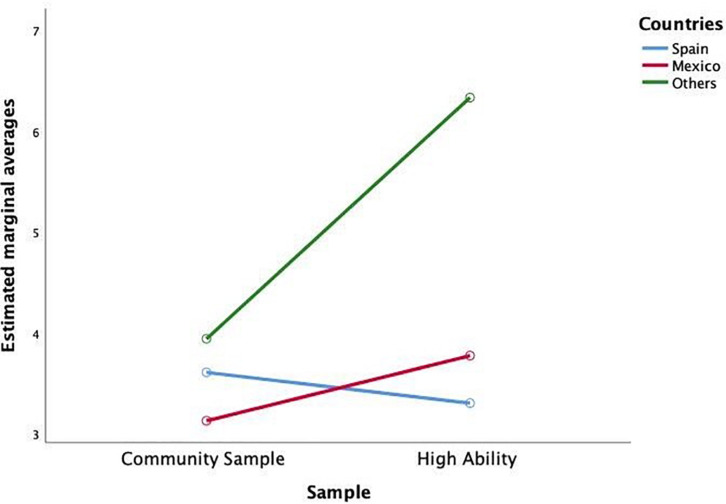
Interaction of the variables country and sample in time devoted to play.

Significant differences are observed in play, both for interaction and for the main effects. Tukey’s post-contrast shows no significant differences between countries, with significantly less time played by the high ability group (Mean = 0.851; DT = 1.62) in relation to the other sample (Mean = 0.912; DT = 1.70). The interaction (Figure 1) indicates that the American high ability students and the Spanish community sample play more.

Another activity where significant differences are found is in doing homework, depending to the country of residence. Since heteroscedasticity has been proven, the Brown-Forsythe (B-F2, 69.92 = 36.46; *p* > 0.001). The highest average time is in Spain (Mean = 2.48; SD = 2.92), followed by the rest of the American countries (Mean = 0.57; SD = 1.53) and Mexico (Mean = 0.50; SD = 1.50). Tukey’s post-test shows significant differences in the time spent doing homeworking between Spain and Mexico (1.98; *p* > 0.001) and between Spain and the rest of the American countries (1.91; *p* > 0.001).

### Qualitative Analysis

**FIGURE 2:**
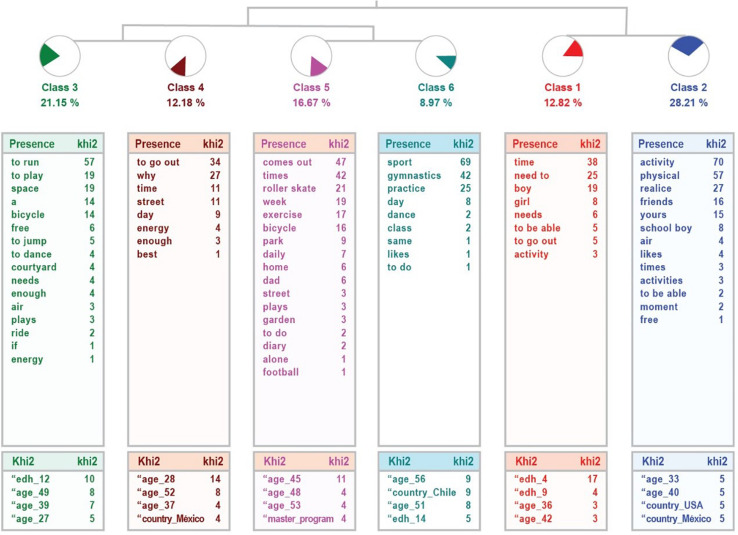
Dendrogram of responses given by parents in the community sample.

In order to determine the perception that parents have about whether or not their children’s physical activity is sufficient, an analysis has been carried out with ALCESTE (Reinert, 2001), separating the two samples. In the case of the parents of the community sample, six classes are obtained (*Need for interaction with peers, Absence of physical activity, Space for physical activity, Going outside, Activity routines and Physical activity at home*), which classify 74% of the textual units and implies a high relevance of the treatment. Figure 2 shows the dendrogram obtained in the analysis. Three links are observed between the classes: the one that links the first with the second (*Need for interaction with peers, Absence of physical activity*), which in turn connects with the others, organized in connection with 3 with 4 (*Space for physical activity, Going outside*), related to the place where the physical activity is done, and 5 with 6 (*Activity routines and Physical activity at home*), which have to do with how the physical activity is done.

Table 11 shows the details of each class: name, number of UCEs it comprises, percentage of variable it explains, most representative word and example sentences of each class.

**TABLE 11:** Information from the analysis to the question “Is the physical activity developed sufficiently or insufficiently?” Community sample.

**Class**		**ECU**	**%**	**Word**
**1. Name**	*Need for interaction with peers*	20	12.82	Time
Phrases	He/she needs to interact with children of his or her age and to strengthen friendship relationships. She needs interaction with other children. She’s an only child. It is necessary to have more time and to be able to go to those places that are proper for children. Children zones, beach, mountain			
**2. Name**	*Lack of physical activity*	44	28.1	Activity
Phrases	He/She is not physically active He/She does not like physical activity He/She does not get too much physical activity			
**3. Name**	*Space for physical activity*	33	21.15	Run
Phrases	We live in an apartment and have no space to run or play freely. Luckily at home there is a very big yard where we can play, run or ride our bikes a little bit like this - which we have made the most of. We go to the farm and run, jump and play.			
**4. Name**	*Going outside*	19	12.8	Exit
Phrases	Because he doesn’t spend enough energy when we go outside Because we can’t go outside. We walk for an hour a day.			
**5. Name**	*Activity routine*	26	16.67	They come out
Phrases	He/She goes out to the garden at least three times a week to exercise with his/her dad. from working out several times a week to sporadic exercise without a daily routine. He/She goes for a walk for 1 h a day and alternates it with biking, rollerblading, skateboarding and at home too.			
**6. Name**	*Physical activity at home*	14	8.97	Sport
Phrases	He usually goes to the gym and now does what he can at home, but we don’t have any machinery (no big place to play sports.) He/She receives online rhythmic gymnastics training routines and does sport with his/her big sister. He/She plays in the room and does sport in the room. Gymnastics.			

**FIGURE 3:**
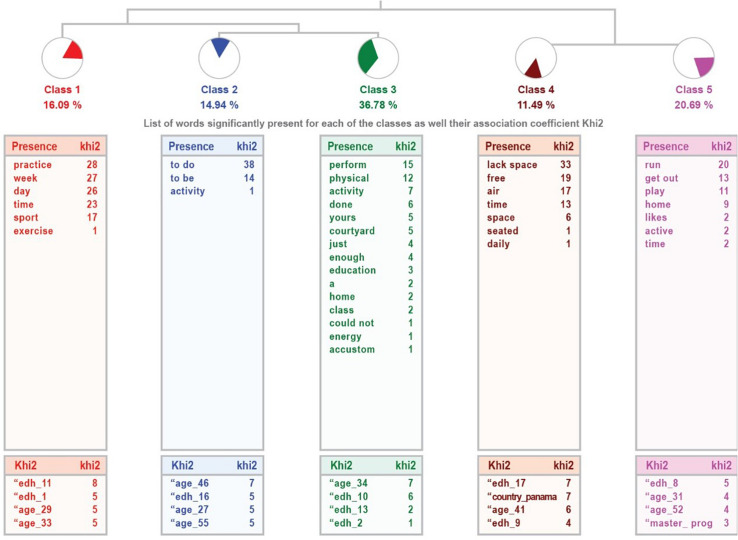
Dendrogram of responses given by parents in the high ability sample.

In the case of the sample of parents with children of high abilities, the analysis extracts five classes (*Practice of physical activity, Little physical activity, Changes in physical activity, Outdoor and Physical activity at home*), which classifies 73% of the textual units, with a high relevance of the treatment. Class 1 (*Practicing physical activity*) links the union of class 2 (*Little physical activity*) with class 3 (*Changes in physical activity*), on the one hand, which are related to the type of physical activity developed, and with class 4 (*Going outside*) and class 5 (*Physical activity at home*), whose content refers to where the physical activity is performed. The dendrogram is shown in Figure 3.

The detail of each class (name, number of UCEs it encompasses, percentage of variable it explains, most representative word and example sentences of each class) is presented in Table 12. Class 1, together with the link between 2 and 3, refers to the practice of physical activity, adapted to confinement, while classes 4 and 5 refer to the place where it is performed.

**TABLE 12:** Information from the analysis to the question “Is the physical activity developed sufficiently or insufficiently?” High ability sample.

**Class**		**ECU**	**%**	**Word**
**1. Name**	*Practice of physical activity*	14	16.09	Practice
Phrases	She practiced several hours of exercise a day, impossible today. We do some sport every day Half an hour of elliptical 7 days a week, and practice the activities marked by the school’s physical education teacher			
**2. Name**	*Little physical activity*	13	14.94	To do
Phrases	Being in confinement he can’t do much sport. He does a 20-min routine of varied exercises. He could do more, but he prefers to spend more time with his friends He’s been known not to exercise			
**3. Name**	*Changes in physical activity*	32	36.78	Produced by
Phrases	He has done daily training that is not as intense as what he does with his coaches, but he has done something. In addition, since they have moderated the children’s outings, we have encouraged them to do activities in movement and not just walk. He has done practically no exercise, neither moderate nor continuous. He started physical activity on Mondays and ended up in a lot of pain. He doesn’t want to get too active. He only follows just dance videos from time to time.			
**4. Name**	*Open air*	10	11.49	Missing
Phrases	He/she needs more time outdoors Because they have space to play and be outdoors. We’re very lucky. We don’t have space for outdoor activities			
**5. Name**	*Activity at home*	18	20.70	Run
Phrases	He/She doesn’t want to leave the house and always plays with legos Now that he can go out he prefers to stay home with his cell phone, computer and TV. They run and play in the garden of their grandparents’ house.			

## Discussion

The results found are in line with demystifying some issues in the high ability students. Firstly, with regard to physical well-being, the high-ability students do not show worse stability than the community samples in these conditions of uncertainty that have resulted from being kept at home due to the confinement caused by the health alert, thus confirming the results found in other studies and under pre-pandemic conditions (Zeidner and Shani-Zinovich, 2011; Olivier et al., 2016). This result also helps to dispel the myth that they establish worse relationships with their peers, and, in general, confirms that no specific problems in personal adjustment or in socio-emotional areas have to be expected in that sample (Borges et al., 2011; Valadez et al., 2015; Egeland, 2019).

With regard to physical activity, the samples are comparable in terms of parents’ perception of the appropriateness of their level of physical activity, since there is no relationship between type of sample and satisfaction with the activity they carried out. These results also go against the fact that high ability students do not enjoy physical activity and confirms the results of Rinn and Wininger (2007) or Hormazábal-Peralta et al. (2018).

The results obtained do not allow affirming that there is a differential perception between the parents of the different countries in the well-being of their children, and or in their level of physical activities during the lockdown due to COVID-19. A significant difference is only observed in gymnastics, which is performed more in America than in Spain.

Regarding the type of activity done during the day, significant differences are observed in two of them. The amount of time dedicated to playing shows a significant interaction both in the type of sample and in the country of residence. Parents perceive that high ability students dedicate more time to play in Spain and in the rest of the American countries, in relation to residents in Mexico.

Significant differences are also found in parents’ perception of time spent on homework, which is significantly higher in Spain. There is no difference in the type of sample. It would be convenient to carry out further studies which would allow us to analyze if, in general, schools in Spain give more homework or if it is an effect which appears only in our study.

On the other hand, in the qualitative analysis carried out with ALCESTE (Reinert, 2001), the perceptions of the families regarding the activity done by the children do not differ too much between the community samples and the high ability ones. In both cases, conditions have forced both a decrease in physical activity and modifications to adapt to the confinement and the community samples affirm miss their friends. The categories obtained in the analysis show this in both samples.

However, parents in the community sample feel that their children need to relate to others, which is not shown in the high-ability sample, which is in line with the findings of Makel et al. (2015) that stressed that the use of time in gifted children could be depending on social and family (as the perceptions of parents) variables and conditions. In line with the arguments of Vidosavljević and Krulj-Drasković (2013) and Lutostanski (2018), the family perceptions of the children’s need seems to be relevant in our results, where parents of the gifted do not seem feeling more concerned about insufficient social activities in the children compared to time the devoted to home schooling.

The limitations of this study coincide with its greatest strength: it has been carried out in an atypical situation, albeit one of a universal nature: the mobility limitations forced by the health alarm caused by the COVID-19 pandemic. On the one hand, these findings are limited to these conditions. But, on the other hand, it is important to know how children have been able to cope with this difficult reality. These special moments we have experienced allow us to analyze how they have handled the situation and fortunately, as a group, neither the community sample nor the high ability sample’s psychological well-being seems to have been affected in a significant way.

Another limitation found is that the reporting of physical activity by the children varies greatly, as can be seen from the great variability shown by the SD values, which means that these results must be taken with caution. In fact, family conditions, their context and the facilities they may or may not have in their home condition their mobility, but especially for the minors.

Finally, although the results obtained do not favor the myth of less physical activity in high-ability students, it is necessary to increase the number of studies, under normal conditions, in order to respond to this issue with greater precision.

What is essential is to continue investigating the real characteristics of high-abilities students in a rigorous way, since this is the appropriate way to know their strengths and weaknesses, so that the best lines of action can be clearly recommended, especially those related to educational intervention.

## Conclusion

Despite the fact that both children and adolescents are a vulnerable population, these results show adequate psychological well-being. On the other hand, despite the limitations, they have engaged in physical activity during confinement, although there is a wide diversity in the number of hours per week they spend on such activity.

When comparing the two samples studied in the research, no significant difference between them appears. This result supports the adequate personal adjustment of these children and adolescents, it being another evidence against the myth of personal and social maladjustment of gifted people and high intellectual abilities.

On the other hand, there are no differences in the physical activity done by both groups of children. With regard to the distribution of time in their daily routine, Spanish parents seem to perceive that their children with high abilities spend less time playing than American parents, and, in both samples, parents residing in Spain also consider that they have done more homework during the day.

How parents have perceived the adequacy of their children’s physical activity has been analyzed from a qualitative perspective, finding that in both groups (community and high ability samples) their assessments are very similar, except for families in the community sample indicating that they miss their friends.

Regarding the practical implications of this research, it should be noted that it contributes to demystifying high-ability students (Borges et al., 2011; Pérez et al., 2017). The results obtained support the position that it is necessary analyze in a scientific way the prejudices and myths that exist with respect to the group of high capacities. All this will help to have an accurate knowledge of this student and devoid of myths.

In conclusion, this study shows the importance of analyzing the differences between high ability students and community samples, in order to establish the aspects that really differentiate between groups, which is especially important when establishing educational responses according to their needs.

## Data Availability Statement

The original contributions presented in the study are included in the article/supplementary material, further inquiries can be directed to the corresponding author/s.

## Ethics Statement

The studies involving human participants were reviewed and approved by Commission of Ethics of Research and Animal Welfare of the University of La Laguna (CEIBA2020-0396). The patients/participants provided their written informed consent to participate in this study.

## Author Contributions

ER-N, DC-S, GL-A, MV, TA, and ÁB have participated in theoretical review. ÁB, ER-N, TA, DC-S, GL-A, MV, and JF have participated in research design and instrument. ÁB and TA has participated in the data analysis. ÁB, ER-N, DC-S, GL-A, MV, and JF have participated in discussion. ÁB, ER-N, DC-S, GL-A, MV, and JF have participated in the study planning, writing, and revision of the article. All authors contributed to the article and approved the submitted version.

## Conflict of Interest

The authors declare that the research was conducted in the absence of any commercial or financial relationships that could be construed as a potential conflict of interest.

## Footnotes

1 http://estadisticas.mecd.gob.es/EducaDynPx/educabase/index.htm?type=pcaxis&path=/Educacion/Alumnado/Apoyo/Curso18-19/AltasCapacidades&file=pcaxis&l=s0
